# Glenoid Radiolucent Lines and Subsidence Show Limited Impact on Clinical and Functional Long-Term Outcomes After Anatomic Total Shoulder Arthroplasty: A Retrospective Analysis of Cemented Polyethylene Glenoid Components

**DOI:** 10.3390/jcm14197058

**Published:** 2025-10-06

**Authors:** Felix Hochberger, Jonas Limmer, Justus Muhmann, Frank Gohlke, Laura Elisa Streck, Maximilian Rudert, Kilian List

**Affiliations:** 1 Department of Orthopaedic Surgery, Julius-Maximilians University Wuerzburg, Koenig-Ludwig-Haus, Brettreichstrasse 11, 97074 Wuerzburg, Germany; felix.hochberger@klh.de (F.H.); jonas.limmer@klh.de (J.L.); justus.muhmann@gmx.de (J.M.); f-gohlke@t-online.de (F.G.); maximilian.rudert@klh.de (M.R.); 2 Department for Shoulder and Elbow Surgery, Rhoen Klinik, 97616 Bad Neustadt/Saale, Germany; 3 Department of Trauma and Reconstructive Surgery, Eberhard-Karls-University Tuebingen, BG Trauma Center Tuebingen, Schnarrenbergstrasse 95, 72076 Tuebingen, Germany; laura.e.streck@gmail.com

**Keywords:** anatomic shoulder arthroplasty, osteolysis, radiographic analysis, functional outcome, implant positioning

## Abstract

**Background**: Glenoid radiolucenct lines (gRLL) and glenoid component subsidence (gSC) after anatomic total shoulder arthroplasty (aTSA) have traditionally been linked to implant loosening and functional decline. However, their impact on long-term clinical outcomes remains unclear. This study aimed to evaluate whether gRLL and gSC are associated with inferior clinical or functional results in patients without revision surgery. **Methods**: In this retrospective study, 52 aTSA cases (2008–2015) were analyzed with a minimum of five years of clinical and radiographic follow-up. Based on final imaging, patients were categorized according to the presence and extent of gRLL and gSC. Clinical outcomes included the Constant-Murley Score, DASH, VAS for pain, and range of motion (ROM). Radiographic parameters included the critical shoulder angle (CSA), acromiohumeral distance (AHD), lateral offset (LO), humeral head-stem index (HSI), and cranial humeral head decentration (DC). Group comparisons were conducted between: (1) ≤2 vs. 3 gRLL zones, (2) 0 vs. 1 zone, (3) 0 vs. 3 zones, (4) gSC vs. no gSC, and (5) DC vs. no DC. **Results**: Demographics and baseline characteristics were comparable across groups. Functional scores (Constant, DASH), pain (VAS), and ROM were largely similar. Patients with extensive gRLL showed reduced external rotation (*p* = 0.01), but the difference remained below the MCID. Similarly, gSC was associated with lower forward elevation (*p* = 0.04) and external rotation (*p* = 0.03), both below MCID thresholds. No significant differences were observed for DC. **Conclusions**: Neither extensive gRLL nor gSC significantly impaired long-term clinical or functional outcomes. As these radiographic changes can occur in the absence of symptoms, regular radiographic monitoring is essential, and revision decisions should be made individually in cases of progressive bone loss.

## 1. Introduction

Anatomic total shoulder arthroplasty (aTSA) is an established treatment for degenerative glenohumeral joint disorders, consistently providing pain relief and functional improvement across diverse patient populations [1]. Long-term follow-up studies report implant survival rates exceeding 90% at ten years, underlining the procedure’s durability and reliability [2]. However, aseptic loosening remains the primary cause of implant failure and revision surgery after aTSA [3].

Radiolucent lines (RLL)—particularly at the cement–bone or implant–bone interface—are regarded as radiographic indicators of potential loosening and may develop around both the humeral and glenoid components [4,5]. Extensive gRLL have been associated with symptomatic loosening and represent a common indication for revision [3,4,6] In contrast, minor lucencies of <2 mm without signs of implant migration, are commonly neglected [3]. They typically appear early in the postoperative course and tend to remain stable over time, rarely causing cliical symptoms or resulting in implant loosening [7].

In addition to gRLL, gSC—defined as migration of the glenoid implant relative to its original position within the scapular bone [7]—has emerged as a radiographic indicator of component loosening. Several studies suggest that micromotion at the implant–cement or cement–bone interface represent an early failure mechanism [7,8]. This process may remain radiographically stable for extended periods but carries the risk of gRLL and gSC over time.

Previous studies investigating gRLL and glenoid implant migration have often been limited by short follow-up durations, heterogeneous implant systems, or a lack of correlation with validated clinical endpoints [6,7,9,10]. Furthermore, inconsistencies in defining gRLL and gSC complicate comparison across studies and limit their utility in guiding treatment decisions in asymptomatic patients.

Given these limitations, there is a need to evaluate the impact of extensive gRLL and gSC in patients with non-revised implants on long-term clinical and functional outcomes.

The aim of this study was to assess the association between the radiographic findings and long-term clinical and functional outcomes in patients treated with cemented all-polyethylene glenoid and stemmed, short-stem or stemless aTSA. By contextualizing gRLL and gSC in relation to implant design and validated outcome scores, this study seeks to inform individualized follow-up and revision strategies in aTSA patients.

The authors hypothesized that radiographically confirmed gRLL and/or gSC would be associated with inferior long-term clinical and functional outcomes following aTSA, with the most pronounced differences expected in patients exhibiting three gRLL zones (extensive gRLL) compared to those with none or only one to two zones.

## 2. Materials and Methods

### 2.1. Study Population

This retrospective study included patients who underwent aTSA at a high-volume tertiary arthroplasty center between January 2008 and December 2015. The primary objective was to evaluate the long-term clinical and functional implications of gRLL and gSC.

Eligible patients were adults (≥18 years) with primary aTSA, who had completed a minimum of 60 months of standardized clinical and radiographic follow-up. Patients who underwent revision surgery at any point during the follow-up period were excluded from the analysis. Implant selection evolved over the study period: cemented stemmed designs with keeled glenoid components were predominantly used in the earlier years, while stemless implants with new-generation all-polyethylene glenoids became standard of care in later years, reflecting advances in implant design and fixation strategies.

Exclusion criteria included any prior shoulder surgery on the affected side, perioperative infection, malignant or vascular disease, or revision procedures during the follow-up period.

Patients were stratified into subgroups based on the presence and extent of gRLL and gSC, as determined from standardized radiographic evaluation at final follow-up. In this study, gRLL was defined as osteolysis exceeding 2 mm in any glenoid zone [6]. Given that the most pronounced differences in clinical outcomes were expected between patients with (1) ≤2 versus 3 affected glenoid zones, this cutoff was used as the primary grouping criterion. Since the presence of radiolucent lines in all three zones is generally considered to carry the highest risk for implant failure, we defined involvement of all three zones in this study as extensive gRLL. In addition, further group comparisons were conducted for (2) 0 vs. 1 gRLL zone, (3) 0 vs. 3 gRLL zones, (4) presence vs. absence of gSC, and (5) presence vs. absence of DC.

### 2.2. Radiographic and Clinical Evauation

Radiographic and implant-related parameters were assessed using standardized pre- and postoperative imaging. At final follow-up, clinical and functional outcomes were systematically evaluated using the Constant-Murley Score, the Disabilities of the Arm, Shoulder and Hand (DASH) index, the visual analog scale (VAS) for pain, and measurements of active range of motion (ROM) in abduction, external rotation, and forward elevation.

Baseline characteristics—such as age, sex, ASA score, BMI, smoking status, and major comorbidities (e.g., osteoporosis, diabetes mellitus, rheumatoid arthritis)—were also recorded.

Radiographic, functional and clinical outcome measures were compared across subgroups to determine the potential association between gRLL, gSC, and long-term clinical or functional impairment.

The study was conducted in accordance with the Declaration of Helsinki and approved by the Institutional Review Board of the Julius-Maximilians University Wuerzburg (Reference No. 20201111 01). Written informed consent was obtained from all participants. Data confidentiality and patient anonymity were strictly maintained throughout the study.

### 2.3. Surgical Technique and Postoperative Care Protocol

All procedures were performed by senior orthopedic surgeons with extensive experience in shoulder arthroplasty. A standardized deltopectoral approach was used in all cases. Subscapularis management was conducted consistently using a transosseous double-row repair technique. Preoperative planning was based on comprehensive imaging, including standard radiographs, magnetic resonance imaging (MRI), and computed tomography (CT) scans, to ensure optimal implant selection and surgical strategy.

Implant usage evolved over the study period:

Until September 2012, cemented stemmed implants (Aequalis™ Cemented, Tornier, Montbonnot-Saint-Martin, France) or head resurfacing prostheses (Copeland™ Surface Replacement Arthroplasty, Zimmer Biomet, Warsaw, IN, USA) were used in combination with a cemented all-polyethylene keeled glenoid component (Aequalis™ Glenoid, Tornier, Montbonnot-Saint-Martin, France). Between October 2012 and December 2013, a stemless implant (Eclipse™ Total Shoulder Prosthesis System, Arthrex, Inc., Naples, FL, USA) was implanted together with a cemented all-polyethylene Keel Glenoid^®^ component (Arthrex, Inc., Naples, FL, USA). Since January 2014, either a stemless implant (Simpliciti™ Shoulder System, Stryker, Inc., Kalamazoo, MI, USA) or a cemented short-stem implant (Aequalis™ Ascend Flex Convertible Shoulder System, Stryker, Inc., Kalamazoo, MI, USA) has been used, both in combination with a cemented all-polyethylene keeled glenoid component (Perform™ Glenoid, Stryker/Tornier, Montbonnot-Saint-Martin, France). Despite generational and instrumentation differences, the basic fixation principles and design features of the glenoid components remained comparable throughout the study period.

Intraoperative data, including cementation technique and the occurrence of any procedure-related complications, were carefully recorded.

Postoperatively, all patients followed a standardized rehabilitation protocol. The shoulder was immobilized in a sling for six weeks, with external rotation strictly limited during this time. Only passive and assisted active movements were permitted in this initial phase. Starting in week seven, patients progressed to active mobilization and gradual strengthening exercises. A full return to sports activities was typically permitted after approximately four months, depending on individual recovery and functional progress.

### 2.4. Radiographic Assessment

**Figure 1:**
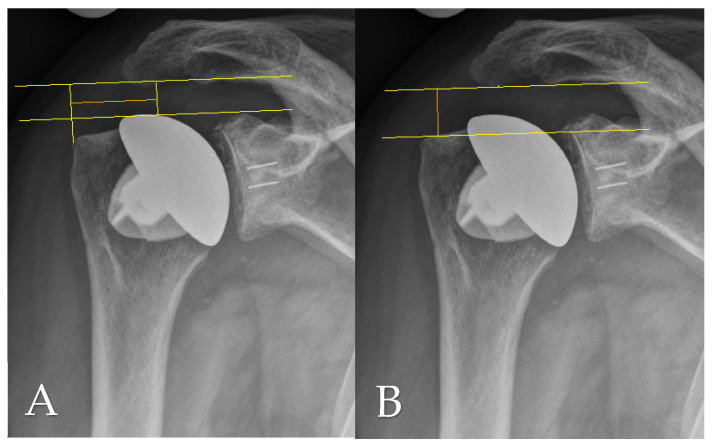
(**A**) Lateral offset (LO; orange line) and (**B**) acromiohumeral distance (AHD; orange line) measured on an anteroposterior radiograph at final follow-up.

**Figure 2:**
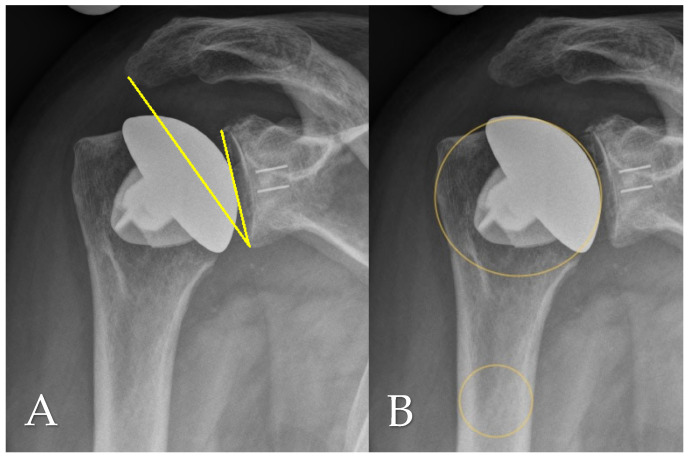
(**A**) Critical shoulder angle (CSA; yellow line) and (**B**) humeral head-stem-index (HSI; yellow circles) measured on an anteroposterior radiograph at final follow-up.

Radiographic evaluation at final follow-up was based on standardized anteroposterior and scapular Y-view radiographs, which were also consistently obtained during routine follow-up assessments. Postoperative imaging was used to analyze several anatomical and implant-related parameters, including the critical shoulder angle (CSA) as per Moor et al. [11], the acromiohumeral distance (AHD), lateral offset (LO), the head–stem index (HSI), and the extent of glenoid erosion following the classification systems proposed by Sirveaux [12], Lévigne, and Franceschi [13]. AHD was determined by measuring the vertical distance between the lateral aspect of the acromion’s undersurface and a horizontal line aligned with the upper border of the greater tuberosity, in accordance with the method described by Berthold et al. [14] (see Figure 1B). The CSA was calculated using lines drawn from the superior to inferior margins of the glenoid and from the inferior glenoid pole to the lateralmost point of the acromion, following Moor’s protocol [11] (Figure 2A). Lateral offset was assessed on true AP views by calculating the horizontal distance from the humeral head center to a vertical reference line extending from the lateral acromion margin to the humeral shaft (Figure 1A). The head–stem index (HSI) was computed as the ratio between the diameter of the humeral head and the shaft (stem) diameter, as outlined by Hochberger et al. [15] (Figure 2B). Assessment of glenoid erosion was conducted on AP radiographs and classified according to the established schemes of Sirveaux, Lévigne, and Franceschi, which consider both the magnitude of bone loss and the degree of medial displacement of the joint line relative to the scapular anatomy.

**Figure 3:**
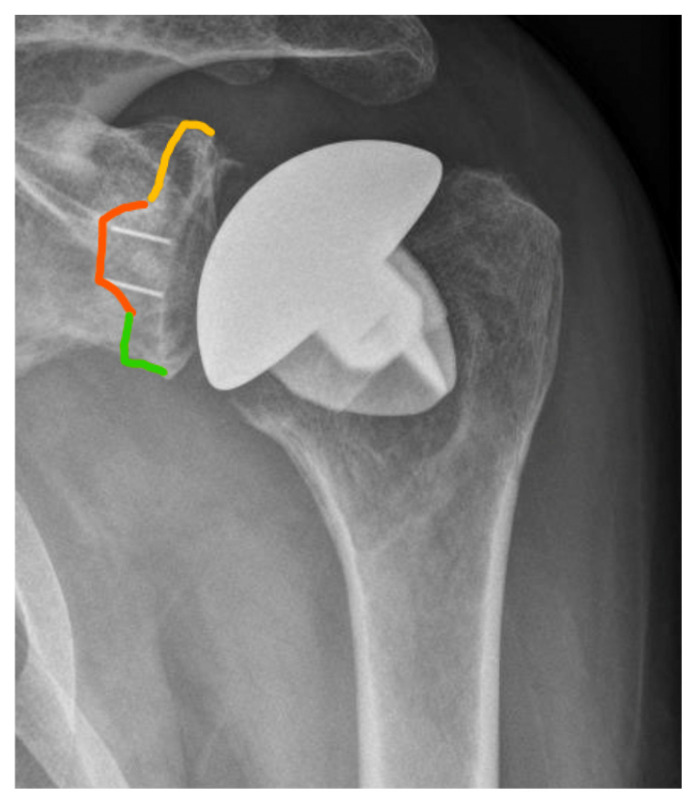
Representative AP view showing radiolucent zones at the cement–bone interface of the glenoid component. Based on the method by Streck et al. [6], the glenoid face was divided into three anatomical thirds: superior (yellow), medial (orange), and inferior (green).

**Figure 4:**
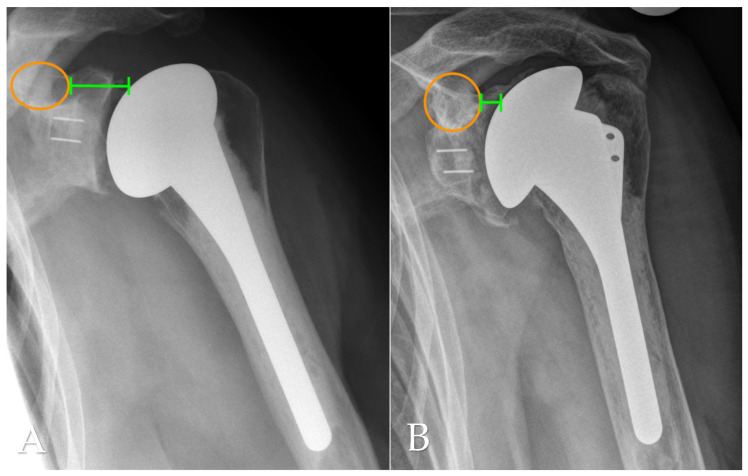
Anteroposterior (AP) radiographs illustrating glenoid component subsidence (gSC). (**A**): Immediate postoperative implant positioning. The orange circle highlights the base of the coracoid process, and the green spacing measurement indicates the distance to the humeral component. (**B**): Final follow-up x-ray showing vertical migration of the gSC, with a reduced distance between the coracoid process and the humeral component.

In the present study, only radiolucent lines >2 mm at the cement–bone interface of the glenoid component were defined as gRLL and included in the analysis, in accordance with established radiographic thresholds [6]. Thin radiolucent lines ≤2 mm without evidence of component migration are frequently observed in clinical practice but were not classified as gRLL in this study. These minor changes have been shown to persist over time without leading to clinical symptoms or implant loosening [3]. For standardized localization, gRLL at the cement–bone interface of the glenoid component were classified according to the system by Streck et al. [6]. This divides the glenoid face into three anatomical thirds (superior, medial, inferior) based on the visible distribution of gRLL on standard AP radiographs (Figure 3). The extent of gRLL was quantified by counting the number of affected zones. Patients were subsequently classified according to the extent of gRLL, with extensive gRLL defined as involvement of 3 zones. GSC was defined as migration of the glenoid component within the bone relative to its initial postoperative position. This was determined through comparative analysis of standardized true AP radiographs taken immediately postoperatively and at the final follow-up (Figure 4).

All radiographic measurements were independently assessed by two specialists (KL and FH). Discrepancies were resolved by consensus review.

### 2.5. Data Analysis and Statistical Methods

Statistical analyses were conducted using SPSS software (Version 27, IBM Corp., Armonk, NY, USA). Continuous data were reported either as means with standard deviations (SD) or as medians with interquartile ranges (IQR), depending on their distribution characteristics. Categorical variables were summarized using both absolute counts and percentages. To evaluate the distribution of continuous variables, the Kolmogorov–Smirnov test was applied. Group comparisons were carried out using independent *t*-tests for normally distributed data, while the Mann–Whitney U test was applied to non-normally distributed variables. Categorical data were analyzed using the chi-square test or Fisher’s exact test, depending on expected frequencies. Group comparisons focused on demographic characteristics, follow-up radiological parameters, and functional outcomes assessed via the Constant and DASH scores. Due to the retrospective nature of the study, a priori sample size calculation was not feasible. To ensure statistical robustness, post hoc power analyses were performed, demonstrating sufficient sensitivity to detect clinically relevant effect sizes (>0.8). For all comparisons, statistical significance was defined as *p* < 0.05.

## 3. Results

### 3.1. Patient Demographics

**Figure 5:**
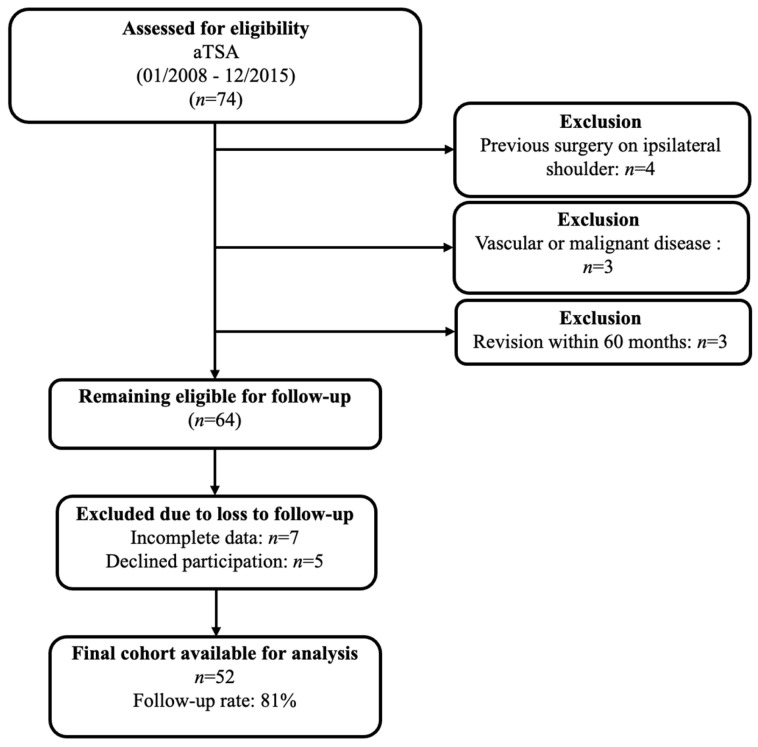
Flowchart illustrating patient selection and exclusion criteria.

A review of the institutional database identified 74 patients who had received an aTSA between January 2008 and December 2015. After applying the exclusion criteria—namely prior ipsilateral shoulder surgery (*n* = 4), vascular or malignant conditions (*n* = 3), and revision procedures within the first 60 months (*n* = 3)—a total of 64 patients remained eligible for follow-up. Of these, 7 were excluded due to incomplete clinical or radiographic data, and 5 declined participation, yielding a final cohort of 52 patients available for long-term evaluation (81% follow-up rate, see Figure 5).

**Figure 6:**
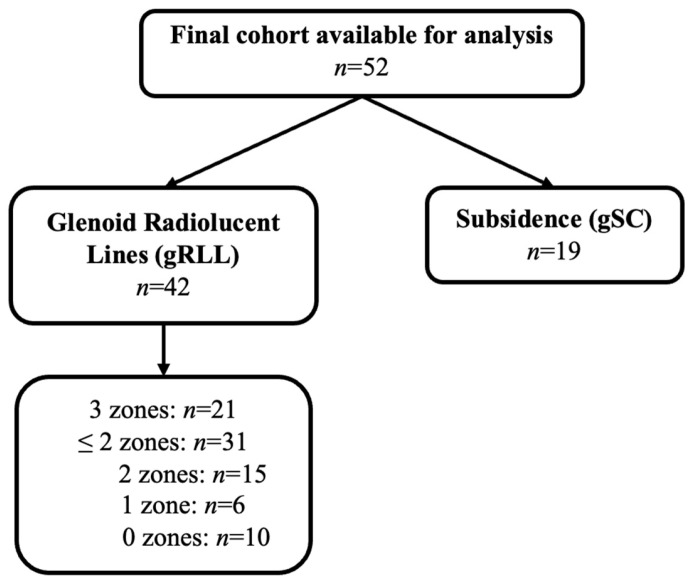
Flowchart illustrating final group allocation based on the presence and extent of gRLL and gSC.

Radiographic evaluation revealed gRLL in 42 of the 52 patients. Within this subgroup, 21 patients showed gRLL in all three defined zones, 15 patients in two zones, and 6 patients in one zone, while 10 patients presented with no detectable gRLL at final follow-up, as illustrated in Figure 6. For statistical analysis, the cohort was primarily divided into patients with involvement of two or fewer zones (*n* = 31) and those with involvement of all three zones (*n* = 21). In addition, gSC was observed in 19 patients across the cohort. An overview of the glenoid component distribution and associated occurrence of gRLL zones and gSC is provided in Table 1.

The distribution of humeral implant types differed between subgroups, with stemmed implants used in 13 of the 21 patients with gRLL in all three zones, compared to 17 stemmed implants among the 31 patients with involvement of up to two zones. This difference was not statistically significant (*p* = 0.61). Due to the small subgroup sizes, no statistical analysis was performed to assess potential correlations between glenoid type and gRLL/gSC occurrence. However, no statistically significant differences were observed in the distribution of individual glenoid types between the groups (Table 2).

There were no significant differences between the two groups regarding age at the time of surgery (62.2 ± 8.0 vs. 62.4 ± 6.9 years; *p* = 0.76), sex distribution (13M/18F vs. 10M/11F; *p* = 0.69), or laterality (right/left: 12/19 vs. 12/9; *p* = 0.19). Similarly, no significant differences were observed in ASA classification (2.1 ± 0.5 vs. 2.4 ± 0.7; *p* = 0.14), BMI (29.4 ± 5.4 vs. 31.2 ± 6.5; *p* = 0.31), or the presence of comorbidities such as osteoporosis, rheumatoid arthritis, diabetes mellitus, and smoking status (all *p* > 0.30). The mean follow-up duration was comparable between groups (122.1 ± 32.8 vs. 121.9 ± 40.0 months; *p* = 0.77). The primary indication for aTSA was primary osteoarthritis (OA) in 31 of 31 patients (100%) in the ≤2 gRLL zone group and in 19 of 21 patients (90.5%) in the 3 gRLL zone group, whereas humeral head necrosis (HN) was present in 2 patients (9.5%). This difference was not statistically significant (*p* = 0.06). A detailed summary of patient demographics and baseline characteristics is provided in Table 3.

### 3.2. Evaluation of Clinical and Functional Results

**Figure 7:**
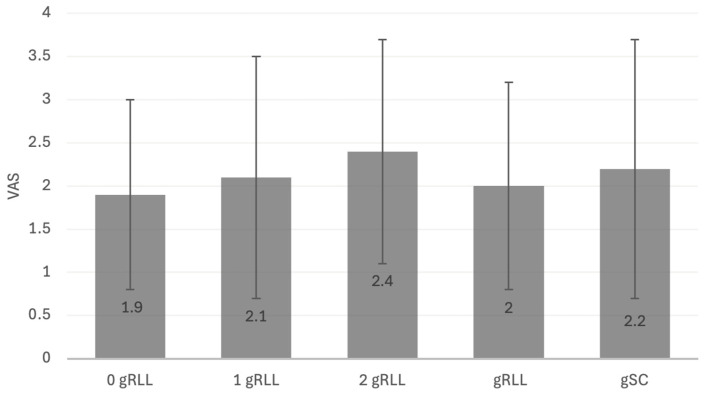
Boxplot analysis of patient-reported pain perception (VAS) across five subgroups stratified by gRLL zones and presence of gSC: 0 gRLL, 1 gRLL, 2 gRLL, 3 gRLL, and gSC. No statistically significant differences in pain levels were observed between the groups (*p* > 0.05), indicating that the extent of gRLL or the presence of gSC does not correlate with increased pain perception at long-term follow-up.

Clinical and functional outcomes were assessed at final follow-up and analyzed across predefined subgroups. To compare cases with incomplete with complete extend of gRLL, subgroups of patients with ≤2 versus those with 3 zones were formed. Subgroup analyses were additionally performed for patients with 0 vs. 1 zone, 0 vs. 3 zones, as well as the presence vs. absence of gSC. Patients with 3 gRLL zones exhibited significantly reduced external rotation compared to those with ≤2 zones (44° ± 4° vs. 47° ± 4°; *p* = <0.05). However, this difference did not exceed the minimal clinically important difference (MCID) threshold of approximately 11° and was therefore considered not clinically relevant. All other ROM parameters and functional outcome scores did not differ significantly between groups. When comparing 0 vs. 1 zone, and 0 vs. 3 zones of gRLL, no significant differences were observed for any clinical outcome measures. In the analysis stratified by presence vs. absence of gSC, patients with gSC showed significantly lower forward elevation (113° ± 14° vs. 123° ± 12°; *p* = <0.05) and external rotation (38° ± 13° vs. 47° ± 16°; *p* = <0.05). As with gRLL, these differences were statistically significant, but did not exceed MCID thresholds, and thus were not regarded as functionally meaningful. No significant differences were found in Constant or DASH scores. Across all subgroup analyses—including comparisons of 0 vs. 1 zone, ≤2 vs. 3 zones, 0 vs. 3 zones, and gSC vs. no gSC—no statistically significant differences in VAS scores were observed (Figure 7). Pain levels remained low across all groups, typically ranging between 1.9 and 2.2 points. DC assessed in an exploratory subgroup analysis, was not significantly associated with differences in clinical or functional outcomes. A detailed overview of all outcome parameters across the respective subgroups is provided in Table 4, Table 5, Table 6, Table 7 and Table 8.

### 3.3. Radiographic Outcomes

Radiographic assessments at final follow-up revealed no significant differences between patients ≤2 gRLL zones and 3 gRLL zones. CSA was comparable between groups (33 ± 7° vs. 31 ± 6°; *p* = 0.19), as were the lateral offset (9 ± 8 mm vs. 12 ± 8 mm; *p* = 0.10) and AHD (8.5 ± 4.6 mm vs. 10.5 ± 4.4 mm; *p* = 0.13). Similarly, HSI (0.4 ± 0.0 vs. 0.4 ± 0.0; *p* = 0.95), Lévigne classification (1.6 ± 0.6 vs. 1.5 ± 0.6; *p* = 0.53), and Sirveaux classification (0.9 ± 0.9 vs. 0.7 ± 0.6; *p* = 0.50) did not differ significantly between groups. These findings suggest that the extent of gRLL was not associated with significant alterations in implant positioning joint alignment (Table 9).

## 4. Discussion

The current study confirmed once more that gRLL (79% of all patients) and even gSC (23% of all patients) are common findings of cemented all-polyethylene glenoids in aTSA at long term follow-up. Interestingly, the study revealed that neither extensive gRLL involving all three zones, nor the presence of gSC of cemented all-polyethylene glenoid in aTSA showed clinically meaningful impact on long-term shoulder function or pain level. No significant correlation in objective shoulder function (ROM, Constant Score), patient-reported outcomes (DASH), or pain perception (VAS) was observed. These findings contradict the initial hypothesis that progressive gRLL and the presence of gSC would translate into worse clinical outcomes over time. On the contrary, patients with advanced radiographic changes achieved long-term outcomes comparable to those without gRLL, suggesting that isolated gRLL or gSC with associated evidence of bone resorption are not necessarily indicative of symptomatic implant failure, even after a mean follow-up exceeding nine years.

Despite radiographically evident changes, long-term clinical and functional outcomes remained stable across all groups. While a slight reduction in abduction and external rotation was observed in patients with gRLL in all three zones compared to those with ≤2 zones, these small differences did not meet the minimal clinically important difference (MCID) thresholds reported in the literature (11–13° for abduction, ~11° for external rotation) [16]. Subgroup analyses confirmed this pattern: neither patients with isolated gRLL in one or two zones nor those with gSC exhibited significantly impaired ROM, functional scores, or pain levels. Even in the most radiographically affected subgroups (3 gRLL zones and gSC), the Constant and DASH scores remained comparable to less affected patients, and VAS scores were virtually unchanged.

These findings challenge the commonly held assumption that progressive radiolucent changes inevitably lead to functional deterioration or clinical failure. The absence of a clear correlation between the extent of gRLL or gSC and shoulder function or pain perception suggests that such radiographic changes may often remain subclinical for extended periods. This is particularly relevant in the context of prior hypotheses regarding early micromotion or ‘pistoning’ within the cement mantle—mechanisms that may lead to radiographic changes without overt clinical symptoms [8,17].

It is important to emphasize that the absence of symptoms should not result in overlooking radiological evidence of loosening. As previous studies [3,4,6,7] have demonstrated, advanced radiographic loosening may eventually translate into pain or implant failure in the even longer term. Of greater significance, however, is the fact that progressive loss of bone stock caused by progressive osteolysis makes revision surgery on the glenoid side exponentially more challenging [18].

Therefore, even in asymptomatic patients with stable shoulder function, the presence of extensive gRLL or gSC warrants closer surveillance and may justify early counseling regarding potential future revision strategies.

Despite the presence of gRLL and gSC in a large proportion of the cohort, no significant differences were observed in key postoperative radiographic parameters—including CSA, AHD, LO and HSI—across the analyzed groups. Even in patients with extensive gRLL or measurable gSC, these parameters remained largely within expected ranges and showed no consistent correlation with functional or clinical impairment.

While decreased AHD and increased HSI have previously been linked to superior humeral migration, rotator cuff dysfunction, and glenoid loosening in aTSA [19,20,21], such associations were not confirmed in our cohort. Our findings are supported by previous studies indicating that moderate variations in CSA, AHD, LO, and HSI do not necessarily translate into functional deterioration, especially when deltoid and rotator cuff function remain intact [15,22]. In addition, analysis of preoperative glenoid morphology revealed a predominance of concentric deformities (Walch A1/A2), which were present in over 80% of cases. In contrast, eccentric glenoid types (Walch B1/B2) were relatively uncommon, representing only a small fraction of the cohort. While such deformities are known to influence implant positioning and fixation mechanics, their distribution was comparable between the groups.

Our data revealed that patients with extensive gRLL were, on average, younger than those with fewer or no radiolucent zones. Although this difference did not reach statistical significance, it contrasts with previous findings that have suggested older age may predispose to periprosthetic osteolysis due to reduced bone quality and impaired remodeling capacity. Experimental studies have demonstrated that aging is associated with inferior trabecular architecture and increased micromotion at the bone–implant interface, particularly in metaphyseal fixation systems such as short-stem or stemless aTSA designs [23].

However, clinical outcome studies indicate that increased age at the time of aTSA does not necessarily compromise long-term results. Su et al. reported comparable improvements in pain and shoulder function among elderly patients undergoing anatomic or reverse TSA, despite a higher prevalence of radiographic changes such as gRLL [10]. Similarly, Crowley et al. found that older patients may even achieve better outcomes than younger cohorts following aTSA, likely due to reduced functional demands and more realistic postoperative expectations [24].

These findings align with our results, demonstrating that neither age nor other baseline characteristics such as ASA classification, BMI, smoking status, or comorbidities (e.g., osteoporosis, diabetes, rheumatoid arthritis) as well as the distribution of surgical indications—namely primary osteoarthritis versus humeral head necrosis—showed a consistent association with the extent of gRLL or the presence of gSC. Thus, while age-related bone quality remains a theoretical risk factor, our data do not support a clear demographic predictor of clinically relevant gRLL or SC after aTSA. Moreover, no significant difference in follow-up duration was observed between the groups (*p* = 0.77), thereby minimizing the risk of bias due to unequal observation times.

Our findings are based on a cohort that included stemless, short-stem and stemmed implants, representing different fixation strategies and biomechanical characteristics on the humeral side. However, as our primary outcome focused on glenoidal changes, and given the existing evidence supporting comparable clinical outcomes across these implant types, it appears highly unlikely whether humeral stem design plays a decisive role in the formation of gRLL on the glenoid side. Interestingly, patients with more extensive gRLL (3 zones) were more likely to have received stemmed implants. This observation suggests a potential link between humeral component design and the development of gRLL. Finite element analyses have demonstrated that stemmed implants tend to reduce cortical bone stress and deviate from the physiological loading pattern, whereas stemless and short-stem designs better preserve native stress distribution in the proximal humerus, potentially mitigating peri-glenoid remodeling [9]. However, this effect, which has so far only been identified on a theoretical level, seems to play a subordinate role. A considerably greater effect seems to result from the evolution of implants during the study period: stemless implants were introduced in combination with newer glenoid components such as the Perform™ keeled glenoid (available from 2014 onwards). By contrast, in earlier practice patients frequently received stemmed cemented designs together with first-generation components like the Aequalis™ keeled polyethylene glenoid. Therefore, the higher rate of gRLL in patients with stemmed implants may at least in part reflect differences in glenoid component generation and fixation techniques, rather than humeral stem design alone. In line, recent high-level studies confirm that humeral fixation type does not independently determine clinical or radiographic outcomes. Wiater et al. demonstrated in a multicenter randomized controlled trial that both stemmed and stemless aTSA implants yield equivalent functional performance and complication rates at two-year follow-up [25]. Similarly, Looney et al., in their systematic review and meta-analysis, found no significant differences in range of motion, revision rates, or patient-reported outcomes between stemmed and stemless aTSA [26].

Taken together, our findings contribute to a more differentiated understanding of gRLL and gSC in aTSA. Rather than serving as direct surrogates of clinical failure, extensive gRLL involving all three glenoid zones and even the presence of gSC did not correlate with inferior clinical or functional outcomes at long-term follow-up. In light of this, isolated radiographic changes—particularly in the absence of symptoms—should be interpreted with caution. Shoulder pain following aTSA may often originate from alternative sources such as rotator cuff pathology, acromioclavicular joint degeneration, or mechanical impingement, which may misleadingly coincide with gRLL on imaging.

Nonetheless, our findings strongly support the implementation of regular long-term radiographic surveillance, even in asymptomatic patients. Extensive gRLL and gSC—while not directly associated with pain or functional decline—can ultimately lead to substantial glenoid bone loss, which significantly complicates potential revision procedures [18].

Therefore, even in the absence of symptoms, patients with radiographic evidence of extensive gRLL or gSC should be carefully counseled, and early revision surgery should be considered on a case-by-case basis to preserve glenoid integrity and optimize long-term outcomes.

The consistently high rates of gRLL (79% of all patients) and gSC (23% of all patients) observed in this study once again highlight the susceptibility of cemented polyethylene components, which represent the Achilles’ heel of aTSA. In an attempt to address this issue, several efforts have already been made with metal-backed glenoids with so far disastrous failure [17,27]. A new concept of metal-backed glenoids with converted bearings (metal liner and PE head) appear to yield more promising outcomes. It will be interesting to observe whether the theoretical advantages of these implants will outweigh the challenges associated with the cemented all-polyethylene glenoids. However, this question can only be answered once comparable long-term studies become available, which will not be expected before the next decade. The present study may contribute valuable data for future comparative research to answer this question.

This study is subject to the inherent limitations of its retrospective design, including potential risks of selection and information bias as well as the absence of advanced imaging modalities. CT scans were only performed selectively in cases of radiographic uncertainty or suspected complications and were not part of routine follow-up. Standard radiographs remain the primary imaging modality for the detection of gRLL, as endorsed by current consensus definitions [2,3,6].

The moderate sample size may have limited statistical power, particularly for subgroup analyses. Although post hoc power analysis confirmed sufficient sensitivity to detect clinically meaningful differences, the absence of statistical significance does not necessarily imply the absence of clinical relevance. In moderately sized cohorts, subtle but relevant effects may go undetected due to type II error.

Furthermore, the study cohort included a heterogeneous mix of implant designs, ranging from stemless and short-stem to stemmed prostheses with cemented fixation. This variability introduces potential confounding, particularly with respect to humeral RLL and overall load distribution. However, key analyses focused on gRLL, which is hardly, if at all, influenced by the humeral fixation strategy.

## 5. Conclusions

GRLL (79% of all patients) and even gSC (23% of all patients) is a common condition of cemented all-polyethylene glenoids in aTSA at long term follow-up. Interestingly, neither extensive gRLL nor the presence of gSC showed clinically meaningful impact on long-term shoulder function or pain level.

The present findings suggest that gRLL and gSC should be interpreted with caution, as they may not represent reliable surrogates of clinical failure. However, due to the risk of progressive bone loss and increasing complexity in revision options, close radiographic follow-up is recommended—even in asymptomatic patients. Revision surgery should be considered on an individual basis in the presence of advanced radiographic changes, even when symptoms are only mild.

## Figures and Tables

**Table 1:** Distribution of gRLL zones and gSC stratified by implant type. Data are presented as absolute numbers with corresponding percentages in parentheses, based on the total number of each implant type. gRLL was categorized according to the number of affected zones (0, 1, 2, or 3). gSC refers to radiographic evidence of vertical migration of the glenoid component. Percentages reflect intra-group proportions.

Glenoid Type	0 gRLL	1 gRLL	2 gRLL	3 gRLL	gSC
Perform™ Glenoid, Stryker/Tornier (*n* = 12)	4 (33%)	1 (8%)	4 (33%)	3 (25%)	3 (25%)
Arthrex Keel Glenoid^®^ (*n* = 4)	0 (0%)	0 (0%)	0 (0%)	4 (100%)	3 (75%)
Aequalis™ Glenoid, Tornier (*n* = 36)	6 (16%)	5 (14%)	11 (31%)	14 (39%)	13 (36%)

**Table 2:** Distribution of glenoid types according to Walch-classification and follow-up duration—A1/A2 vs. B1/B2. Data is presented as absolute frequencies and mean ± standard deviation. Follow-up duration was compared between groups using Welch’s *t*-test due to unequal variances. A *p*-value < 0.05 was considered statistically significant. No B3, C, or D glenoids were observed in this cohort.

Walch Group	≤2 Zones	3 Zones	Total	Mean Follow-Up (m)	*p*-Value
A1/A2	28	15	43	125.5 ± 35.1	0.09
B1/B2	2	7	9	105.3 ± 34.3	0.14

**Table 3:** Patient Demographics. Data are presented as mean ± standard deviation (SD) with corresponding 95% confidence intervals (CI) or absolute numbers. Comparative analyses between groups with ≤2 gRLL and 3 zones were performed using independent *t*-tests, Mann–Whitney U tests, Fisher’s exact test, or Chi^2^-tests as appropriate. Statistical significance was defined as *p* < 0.05. ASA = American Society of Anesthesiologists Physical Status Classification; BMI = Body Mass Index; OA = Osteoarthritis; HN = Humeral Head Necrosis.

Parameter	≤2 Zones (*n* = 31) (Mean ± SD, 95% CI)	3 Zones (*n* = 21) (Mean ± SD, 95% CI)	*p*-Value
Age at surgery (years)	62.2 ± 8.0 (59.2–65.1)	62.4 ± 6.9 (59.3–65.6)	0.76
Sex (male/female)	13 (M)/18 (F)	10 (M)/11 (F)	0.69
Side (right/left)	12 (R)/19 (L)	12 (R)/9 (L)	0.19
Follow-up (months)	122.1 ± 32.8 (110.0–134.1)	121.9 ± 40.0 (103.6–140.0)	0.77
ASA Classification	2.1 ± 0.5 (1.9–2.3)	2.4 ± 0.7 (2.0–2.7)	0.14
BMI	29.4 ± 5.4 (27.5–31.4)	31.2 ± 6.5 (28.3–34.2)	0.31
Osteoporosis (yes/no)	2/29	3/18	0.35
Rheumatoid arthritis (yes/no)	1/30	2/19	0.34
Diabetes mellitus (yes/no)	2/29	3/18	0.35
Smoker (yes/no)	4/27	5/16	0.30
Fixation (cemented/uncemented)	30/1	21/0	0.40
Indication (OA/HN) Implant type (stemless/short-stem)	31/0 14/17	19/2 8/13	0.06 0.61

**Table 4:** Comparison of range of motion (ROM), Constant Score, DASH, and VAS between patients with limited (≤2 zones) and extensive (3 zones) gRLL. Data are presented as mean ± standard deviation and 95% confidence intervals. Statistical comparisons were performed using independent *t*-tests or Mann–Whitney U tests, as appropriate. *p*-values < 0.05 are considered significant. Abbreviations: FE—Forward Elevation, ABD—Abduction, ER—External Rotation, IR—Internal Rotation, VAS—Visual Analog Scale, Constant—Constant-Murley Score, DASH—Disabilities of the Arm, Shoulder and Hand Score. Statistically significant findings (*p* < 0.05) appear in bold.

Parameter	≤2 Zones (*n* = 31) (Mean ± SD, 95% CI)	3 Zones (*n* = 21) (Mean ± SD, 95% CI)	*p*-Value
FE (°)	121 ± 9 (118–124)	118 ± 9 (114–122)	0.27
ABD (°)	114 ± 10 (110–118)	111 ± 7 (108–114)	0.26
ER (°)	47 ± 4 (45–48)	44 ± 4 (42–46)	**<0.05**
IR (°)	34 ± 5 (32–36)	35 ± 6 (32–38)	0.60
VAS (0–10)	2.4 ± 1.3 (1.7–1.5)	2.1 ± 0.9 (1.9–1.4)	0.09
Constant	66 ± 21 (58–74)	64 ± 12 (59–70)	0.78
DASH	34 ± 24 (25–43)	25 ± 20 (15–34)	0.14

**Table 5:** Subgroup analysis comparing key functional parameters between patients with no gRLL and those in one gRLL zone. Data are presented as mean ± standard deviation and 95% confidence intervals. Statistical comparisons were performed using independent *t*-tests or Mann–Whitney U tests, as appropriate. Abbreviations: FE—Forward Elevation, ABD—Abduction, ER—External Rotation, IR—Internal Rotation, VAS—Visual Analog Scale, Constant—Constant-Murley Score, DASH—Disabilities of the Arm, Shoulder and Hand Score.

Parameter	0 Zones (*n* = 10) (Mean ± SD, 95% CI)	1 Zone (*n* = 6) (Mean ± SD, 95% CI)	*p*-Value
FE (°)	121 ± 12 (113–130)	122 ± 11 (111–134)	0.75
ABD (°)	120 ± 7 (115–125)	112.8 ± 12.3 (100–126)	0.23
ER (°)	49 ± 2 (47–50)	46 ± 4 (42–51)	0.33
IR (°)	36 ± 5 (33–39)	33 ± 5 (27–38)	0.19
VAS (0–10)	1.9 ± 1.1 (1.5–1.7)	2.1 ± 1.4 (1.7–1.3)	0.07
Constant	55.4 ± 25.6 (37.0–73.7)	69.7 ± 15.1 (53.8–85.6)	0.33
DASH	45.4 ± 20.9 (30.5–60.4)	24.4 ± 22.5 (0.8–48.0)	0.05

**Table 6:** Analysis of range of motion, pain, and functional scores in patients with no gRLL with pronounced gRLL affecting all three zones. Data are presented as mean ± standard deviation and 95% confidence intervals. Statistical comparisons were performed using independent *t*-tests or Mann–Whitney U tests, as appropriate. Abbreviations: FE—Forward Elevation, ABD—Abduction, ER—External Rotation, IR—Internal Rotation, VAS—Visual Analog Scale, Constant—Constant-Murley Score, DASH—Disabilities of the Arm, Shoulder and Hand Score. Statistically significant findings (*p* < 0.05) appear in bold.

Parameter	0 Zones (*n* = 10) (Mean ± SD, 95% CI)	3 Zones (*n* = 21) (Mean ± SD, 95% CI)	*p*-Value
FE (°)	121 ± 11 (113–124)	118 ± 9 (114–122)	0.44
ABD (°)	114 ± 10 (110–118)	120 ± 7 (115–125)	**<0.05**
ER (°)	48 ± 2 (47–50)	44 ± 4 (42–46)	**<0.05**
IR (°)	35.9 ± 4.5 (33–39)	35 ± 6 (32–38)	0.62
VAS (0–10)	1.9 ± 1.1 (1.5–1.7)	2.0 ± 1.2 (1.7–1.8)	0.07
Constant	55.4 ± 25.6 (37.0–73.7)	64.3 ± 12.4 (58.7–69.9)	0.32
DASH	45.4 ± 20.9 (30.5–60.4)	24.6 ± 20.2 (15.3–33.8)	**<0.05**

**Table 7:** Comparison of functional and radiographic parameters based on the presence or absence of vertical glenoid component migration (SC). Data are presented as mean ± standard deviation and 95% confidence intervals. Statistical comparisons were performed using independent *t*-tests or Mann–Whitney U tests, as appropriate. Abbreviations: FE—Forward Elevation, ABD—Abduction, ER—External Rotation, IR—Internal Rotation, VAS—Visual Analog Scale, Constant—Constant-Murley Score, DASH—Disabilities of the Arm, Shoulder and Hand Score. Statistically significant findings (*p* < 0.05) appear in bold.

Parameter	gSC (*n* = 19) (Mean ± SD, 95% CI)	No gSC (*n* = 33) (Mean ± SD, 95% CI)	*p*-Value
FE (°)	116 ± 10 (112–121)	122 ± 8 (119–125)	**<0.05**
ABD (°)	111 ± 9 (107–115)	114 ± 10 (110–117)	0.40
ER (°)	44 ± 5 (42–46)	46 ± 4 (45–48)	**<0.05**
IR (°)	33 ± 6 (31–36)	35 ± 6 (33–37)	0.27
VAS (0–10)	2.2 ± 1.5 (1.7–2.7)	2.0 ± 0.4 (1.9–2.1)	0.07
Constant	66.0 ± 13.3 (59.6–72.5)	64.7 ± 20.3 (57.5–71.9)	0.80
DASH	22.7 ± 18.6 (13.7–31.6)	34.6 ± 24.3 (25.9–43.2)	0.07

**Table 8:** Comparison of ROM, Constant Score, DASH, and VAS depending on the presence or absence of cranial humeral head decentration on final follow-up imaging. Data are presented as mean ± standard deviation and 95% confidence intervals. Statistical comparisons were performed using independent *t*-tests or Mann–Whitney U tests, as appropriate. *p*-values < 0.05 are considered significant. Abbreviations: FE—Forward Elevation, ABD—Abduction, ER—External Rotation, IR—Internal Rotation, VAS—Visual Analog Scale, Constant—Constant-Murley Score, DASH—Disabilities of the Arm, Shoulder and Hand Score.

Parameter	Decentration (*n* = 19) (Mean ± SD, 95% CI)	No decentration (*n* = 33) (Mean ± SD, 95% CI)	*p*-Value
FE (°)	120 ± 9 (116–124)	120 ± 10 (116–123)	0.96
ABD (°)	112 ± 9 (108–116)	113 ± 9 (110–116)	0.78
ER (°)	44 ± 4 (42–46)	46 ± 4 (45–48)	0.06
IR (°)	34 ± 5 (32–36)	35 ± 6 (33–37)	0.63
VAS (0–10)	1.8 ± 0.8 (1.3–1.4)	2.1 ± 0.7 (1.4–1.1)	0.07
Constant	64.9 ± 18.1 (56.7–73.1)	65.4 ± 18.2 (58.8–72.0)	0.92
DASH	31.0 ± 25.0 (19.6–42.4)	29.7 ± 21.8 (21.7–37.7)	0.85

**Table 9:** Radiological Parameters at Final Follow-up. ≤2 gRLL zones vs. 3 gRLL zones. Data are presented as mean ± standard deviation and 95% confidence intervals. Differences between groups were analyzed using parametric (*t*-test) or non-parametric (Mann–Whitney U) methods, as appropriate. A threshold of *p* < 0.05 was applied to determine statistical significance. CSA = Critical Shoulder Angle; LO = Lateral Offset; AHD = Acromiohumeral Distance; HSI = Head–Stem Index.

Parameter	≤2 Zones (*n* = 31) (Mean ± SD, 95% CI)	3 Zones (*n* = 21) (Mean ± SD, 95% CI)	*p*-Value
CSA°	33 ± 7 (30–36)	31 ± 6 (29–33)	0.19
LO	9 ± 8 (6–12)	12 ± 8 (9–15)	0.10
AHD	8.5 ± 4.6 (6.5–10.5)	10.5 ± 4.4 (8.8–12.2)	0.13
HSI	0.4 ± 0.0 (0.4–0.5)	0.4 ± 0.0 (0.4–0.5)	0.95
Levigne	1.6 ± 0.6 (1.3–1.8)	1.5 ± 0.6 (1.2–1.7)	0.53
Sirveaux	0.9 ± 0.9 (0.6–1.3)	0.7 ± 0.6 (0.5–1.0)	0.50

## Data Availability

Dataset available on request from the authors.
